# Progressive White Matter Changes in Mitochondrial Disease: A Quantitative MRI Study

**DOI:** 10.1002/jmd2.70093

**Published:** 2026-05-08

**Authors:** Nora Mickelsson, Jussi Hirvonen, Mika H. Martikainen

**Affiliations:** ^1^ Clinical Neurosciences, Department of Clinical Medicine University of Turku Turku Finland; ^2^ Neurocenter Turku University Hospital Turku Finland; ^3^ Department of Radiology Turku University Hospital Turku Finland; ^4^ Department of Radiology, Faculty of Medicine and Health Technology University of Tampere Tampere Finland; ^5^ Research Unit of Clinical Neuroscience, Neurology University of Oulu Oulu Finland; ^6^ Neurocenter and Medical Research Center Oulu University Hospital Oulu Finland

**Keywords:** disease progression, longitudinal imaging, mitochondrial disease, quantitative MRI, small‐vessel pathology, white matter hyperintensities

## Abstract

Primary mitochondrial diseases frequently affect the central nervous system, yet the extent, distribution and progression of white matter hyperintensities (WMHs) remain insufficiently characterised, particularly in terms of quantitative volumetrics and longitudinal progression. Although WMHs are typically attributed to cerebral small‐vessel disease, mitochondrial disorders may cause white matter injury through distinct vascular and metabolic mechanisms. We conducted a retrospective single‐centre study at Turku University Hospital including 36 patients with mitochondrial disease, each with at least one brain MRI (73 images). Longitudinal data were available for 15 patients. Three‐dimensional T1‐weighted and FLAIR images (1.5/3 T) were analysed with the FDA‐cleared cNeuro tool to obtain intracranial volume–normalised WMH and lesion volumes and an automated global Fazekas score. At baseline (median age 49 years), WMHs were present in all supratentorial regions. Over time, WMH volumes increased significantly in periventricular, deep and juxtacortical regions, while lesion progression was predominantly periventricular. Fazekas scores remained generally low and stable. In follow‐up imaging, women and patients carrying the m.3243A>G variant showed a greater burden of WMHs and lesions, compared with men and those with other mitochondrial diagnoses. WMH load did not differ according to history of stroke‐like episodes. Mitochondrial disease is associated with early and progressive WMH accumulation, particularly in individuals with the m.3243A>G variant, and the pattern exceeds what would be expected from conventional vascular risk factors alone. These findings support a disease‐specific mechanism of white matter vulnerability and highlight the importance of quantitative MRI for monitoring progression in mitochondrial disease.

## Synopsis

Our findings demonstrate early and progressive white‐matter involvement in mitochondrial disease, most pronounced in patients with the m.3243A>G variant, supporting a disease‐specific mechanism of white‐matter vulnerability.

## 1 Introduction

Primary mitochondrial diseases, caused by pathogenic variants in mitochondrial DNA (mtDNA) or in nuclear genes encoding mitochondrial proteins, impair cellular ATP production. More than 350 disease‐causing variants are now recognised, and the overall prevalence is estimated at 1:4300 [1, 2]. In Southwestern Finland, the adult prevalence of mtDNA disease has recently been estimated at 9.2/100 000, and 4.2/100 000 for mitochondrial disease associated with the common pathogenic mtDNA variant m.3243A>G [3], which often causes the mitochondrial encephalomyopathy, lactic acidosis, and stroke‐like episodes (MELAS) syndrome. Central nervous system manifestations, such as stroke‐like episodes (SLEs) and epilepsy, are common in mitochondrial disease [4, 5]. Typical brain magnetic resonance imaging (MRI) findings include cerebellar‐predominant atrophy, white matter (WM) abnormalities, basal ganglia calcifications and focal abnormalities including stroke‐like lesions (SLLs) [6, 7, 8].

White matter hyperintensities (WMHs) detected on brain T2‐weighted MRI are a radiological manifestation of WM injury and are most often attributed to cerebral small‐vessel disease (CSVD) [9]. Greater WMH burden, particularly higher number and larger size of lesions, is associated with increased risk of stroke, cognitive decline and mortality [10]. However, in addition to CSVD, a wide range of other aetiologies can also cause WMH. These include, for example, genetic leukoencephalopathies and mitochondrial diseases.

Mitochondrial disorders exhibit diverse patterns of WM involvement. Reported abnormalities range from confluent supratentorial WM lesions, sometimes with cystic components, to combined cerebellar, brainstem and basal‐ganglia involvement [11, 12]. Elevated lactate detected on proton MR spectroscopy may further support a mitochondrial aetiology by indicating impaired oxidative metabolism [11, 13].

MRI phenotypes vary widely across mitochondrial genotypes. Diffuse WM injury and global cerebral atrophy are frequently observed in association with the m.3243A>G variant [6, 7]. In contrast, nuclear‐encoded disorders may display syndrome‐specific patterns, such as the continuous corticospinal tract and dorsal column involvement seen in leukoencephalopathy with brainstem and spinal cord involvement and lactate (LBSL) caused by *DARS2* variants [14, 15, 16], or the MS‐like demyelinating lesions sometimes found in Leber hereditary optic neuropathy (LHON) [17]. *POLG*‐related disease often features posterior cortical–subcortical and thalamic signal changes with variable WM involvement, occasionally progressing to laminar necrosis or necrotizing leukoencephalopathy [18, 19, 20]. Diffusion MRI studies additionally demonstrate widespread microstructural WM abnormalities extending beyond visible fast fluid‐attenuated inversion recovery (FLAIR) lesions, suggesting shared bioenergetic and vascular mechanisms across mitochondrial genotypes [21].

Despite these previous reports, understanding of the scope and spectrum of WM changes in mitochondrial disease as well as their progression over time remains limited. Previous studies have mostly been based on qualitative visual assessments of MRI, leaving quantitative and longitudinal data limited. In the present study, we investigated a cohort of mainly adult mitochondrial disease patients to quantify baseline brain WM involvement and its progression over time using normalised brain MRI WMH volumes alongside the Fazekas scores and lesion volume metrics.

## 2 Methods

We conducted a retrospective, observational, single‐centre study utilising the mitochondrial disease patient cohort at Turku University Hospital (TUH) in Southwest Finland. Patients were identified from the electronic medical record (earliest entries from 1 January 2000), and clinical and imaging data collection was completed in 2023. From this cohort, we included all patients with genetically confirmed mitochondrial disease who had at least one available brain MRI suitable for analyses. The study was approved by the institutional board of TUH (research permission TO4/016/16). Given its retrospective, registry‐based design, individual informed consent was waived.

Multiple scanner models were used over the study period. Of the 73 MR images, 33 were performed at 3 T and 40 at 1.5 T. The core protocol included a three‐dimensional T1‐weighted gradient‐echo (3D T1‐w) sequence and a FLAIR sequence. We exported images in DICOM format and analysed them using the cNeuro MR quantification tool (Combinostics) [22]. WMH and lesion volumes were segmented on FLAIR using a multistep expectation–maximisation approach. Volumes used in the statistical analyses were normalised for head size and expressed relative to age‐ and sex‐adjusted normative reference values [23, 24, 25]. We also rated the WMHs using the Fazekas scale (0–3: 0 = *none*, 1 = *punctate*, 2 = *beginning confluence* and 3 = *confluent areas*) [26]. The global Fazekas score provides an individual‐level grading of WMH burden and is widely used in both clinical and research settings, particularly in small‐vessel and neurodegenerative disease cohorts [9]. The cNeuro software is FDA‐cleared and is clinically implemented at TUH.

We used normalised WMH and lesion volumes as primary outcomes in total brain tissue and subregions (periventricular, juxtacortical and deep WM; infratentorial, pons and cerebellum), and the global Fazekas score. Two analysis sets were defined: the first available brain MRI per patient (*n* = 36), and the last available MRI in patients with ≥ 2 MRI (*n* = 15). Subgroup analyses were performed by sex (women/men), genotype (m.3243A>G vs. other mitochondrial diagnoses, including both mtDNA and nuclear DNA variants), and history of SLE (yes/no) or brain symptoms such as ataxia, encephalopathy and epilepsy (yes/no) extracted from clinical data. For patients with ≥ 2 MRIs, change was calculated as the difference (last–first) and annualised by follow‐up time (mL/year for WMH and lesion volumes, points/year for Fazekas score). Percentage change in total WMH and lesion volumes was estimated from group‐level median values using the ratio of follow‐up to baseline measurements. The periventricular compartment was additionally analysed due to showing the largest relative change.

All distributions were right‐skewed and non‐normal (Shapiro–Wilk *p* < 0.05), and therefore we used non‐parametric methods. Results are reported as median [IQR]. Cohort‐level burden vs. zero was tested with the one‐sample Wilcoxon signed‐rank test at baseline, follow‐up and for annualised change. Between‐group comparisons were assessed with the two‐sided Mann–Whitney *U* test, and within‐patient change (first vs. last MRI) with the two‐sided Wilcoxon signed‐rank test. FDR correction was applied to the main longitudinal WMH and lesion volume analyses. Other analyses were considered exploratory and were not subjected to multiple‐comparison correction. Analyses were performed using IBM SPSS Statistics v29. Statistical significance was set at two‐sided *p* < 0.05.

## 3 Results

Among the 76 patients with mitochondrial disease in the cohort at TUH, 36 (15 men, 21 women) had brain MRI data suitable for quantitative analysis, comprising a total of 73 MRI studies. Fifteen patients (42%) had longitudinal imaging data available (2–8 images/patient, median follow‐up 6.0 years, range 0.5–15) (Table 1).

**TABLE 1:** Demographic, clinical and brain MRI characteristics of the mitochondrial disease cohort.

	All (*N* = 36)	m.3243A>G (*N* = 16, 44%)	POLG^a^ (*N* = 2, 6%)	Other mDNA^b^ (*N* = 15, 42%)	Other nDNA^c^ (*N* = 3, 8%)
Sex (F/M), *N* (%)	F, 21 (58) M, 15 (42)	F, 9 (56) M, 7 (44)	F, 2 (100)	F, 8 (53) M, 7 (47)	F, 2 (67) M, 1 (33)
Current age or age at death, median (years) (range)	56 (2–84)	54 (11–79)	25 (20–30)	63 (2–84)	30 (29–40)
History of SLE, *N* (%)	12 (33)	7 (44)	2 (100)	3 (20)	0
Presence of brain symptoms, *N* (%)	15 (42)	7 (44)	2 (100)	6 (40)	0
Age at first brain MRI (years) (range)	49 (0.6–82)	47 (5–66)	20 (17–22)	56 (0.6–82)	10 (10–30)
Brain MRIs/patient, mean (range)	2 (1–8)	2 (1–5)	5 (2–8)	2 (1–4)	1
Interval between first and last brain MRIs, range (years)	0.5–15	0.5–15	3–7	1–8	—
Hypertension (%)	9 (25)	5 (31)	0	4 (27)	0
Hyperlipidaemia (%)	14 (39)	8 (50)	0	6 (40)	0
Diabetes (%)	12 (33)	10 (63)	0	2 (13)	0
Smoking (%)	4 (11)	1 (6)	0	3 (20)	0

Abbreviations: F = female, M = male, MRI = magnetic resonance imaging, mtDNA = mitochondrial DNA, nDNA = nuclear DNA, SLE = stroke like episode.

^a^ W748S homozygous variant in *POLG*.

^b^ m.11778G>A *N* = 6, m.8344A>G *N* = 2, m.8993T>G, m.3271T>C, m.13513G>A, m.10427G>T, m.3260A>G, mtDNA single deletion, axial myopathy with multiple mtDNA deletions, no definitive molecular genetic diagnosis (*N* = 1 in all).

^c^ Compound heterozygous mutations c.228‐20_21delTTinsC (p.R76SfxX5) and c.492+2T>C (p.M134_K185del) in the *DARS2* gene (*N* = 3).

**FIGURE 1:**
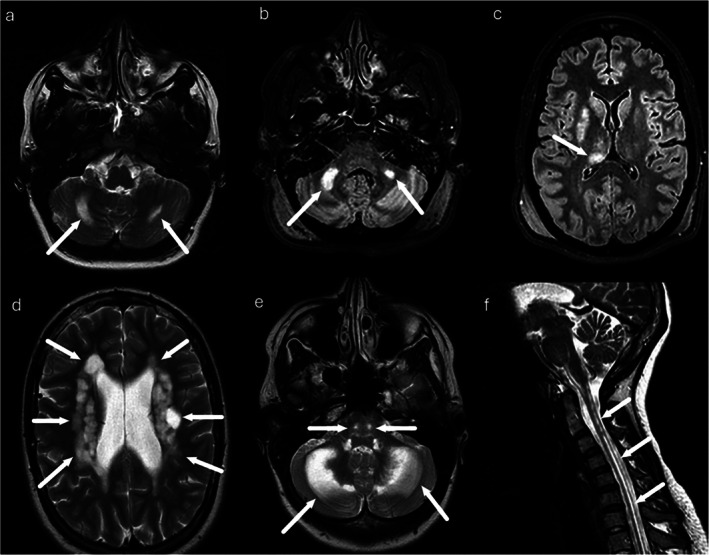
Brain MRI findings related to patients with *POLG*‐related disease and LBSL. (a–c) *POLG*‐related disease in a patient imaged first in 2007 (a; age 22, T2‐weighted) and again in 2014 (b, c; age 29, FLAIR). (a) Bilateral cerebellar WMH were already present years before the first SLE and remained radiologically stable. (b, c) Follow‐up imaging shows persistence of supratentorial WM lesions with the appearance of new abnormalities in the right thalamic region. (d–f) LBSL findings in 23‐year‐old patients (T2‐weighted). Demonstrating the characteristic extensive supra‐ and infratentorial white matter abnormalities, including involvement of the cerebellar white matter, dorsal brainstem pathways, deep nuclear structures and the cervical spinal cord.

The most frequent molecular cause of mitochondrial disease was the m.3243A>G mutation in mtDNA (*n* = 16). Nuclear DNA variants included the homozygous p.W748S variant in *POLG* (*n* = 2) and compound heterozygous mutations c.228‐20_21delTTinsC (p.R76SfxX5) and c.492+2T>C (p.M134_K185del) in *DARS2* in all three patients (*n* = 3). Two patients harbouring *POLG* variants had longitudinal imaging available. In one case, cerebellar WMHs were already present several years before the first SLE and remained radiologically stable thereafter (Figure 1). Three patients carrying pathogenic *DARS2* variants consistent with LBSL had no follow‐up imaging. Their baseline MRIs demonstrated the characteristic extensive supra‐ and infratentorial WM abnormalities (Figure 1).

The earliest MRI was at a median age of 49 years (range 0.6–75 years), and the last available imaging at a median age of 56 years (range 9–81 years). Median ages were similar in the m.3243A>G subgroup (47 and 56 years). At baseline, women were slightly older than men (median 51 vs. 45 years; *p* = 0.5), and at follow‐up, the same age difference remained (median 62 vs. 51 years; *p* = 0.2). Twelve patients (33%) had a history of at least one SLE; these patients have been reported in detail elsewhere [27]. At baseline, 15 patients had a history of some brain symptoms. A subset of patients had conventional vascular risk factors: hypertension in nine (25%), hyperlipidaemia in 14 (39%), diabetes in 12 (33%) and smoking in four (11%) (Table 1).

In baseline MRI (*n* = 36) the median normalised WMH total volume was 6.37 [IQR 24.06] ml. Distributions were right skewed with wide dispersion: deep WM showed the highest median 3.21 [16.41], followed by periventricular 0.50 [5.00] and juxtacortical 0.15 [0.63] regions. Infratentorial WMHs were minimal or absent. One‐sample Wilcoxon tests against zero indicated cohort‐level WMH burden above zero in all regions. Normalised lesion volume was median 1.82 [18.52] ml (by region: periventricular 0.69 [16.69], deep WM 0.42 [0.63] and juxtacortical 0.14 [0.53]). Infratentorial lesion burden was again low or absent. In baseline group comparison (WMH and lesion volumes), no statistical differences were detected by sex, SLE in history, or brain symptoms.

In the m.3243A>G subgroup (*n* = 16), baseline WMH and lesion volumes were comparable, with a tendency towards higher values in total and deep WM. Median WMH total volume was 7.99 [19.77] mL (deep 3.50 [14.92], periventricular 0.70 [3.39], juxtacortical 0.12 [0.48]), with minimal infratentorial involvement. Lesion volumes showed a similar pattern (total 2.30 [11.51], periventricular 0.90 [9.10], deep 0.44 [1.54], juxtacortical 0.12 [0.52]) and Fazekas scores were low (0.013 [0.16]).

In follow‐up imaging (*n* = 15), the total WMH volume was 12.57 [28.58] mL; by region, periventricular 4.41 [6.61], juxtacortical 0.97 [2.81] and deep WM 5.68 [26.85]. The total lesion volume was 9.08 [29.37] mL; periventricular 8.83 [27.20], deep WM 0.59 [1.79] and juxtacortical 0.31 [1.34]. Infratentorial burden remained minimal in both WMH and lesion analyses. Paired comparisons from baseline to the last available image showed significant WMH increases in periventricular (*p* = 0.003), juxtacortical (*p* = 0.005) and deep WM (*p* = 0.005) regions (Table 2). Lesion volume increased significantly in total brain WM and in periventricular compartments (both *p* < 0.001). The main WMH and lesion progression findings remained significant after FDR correction. Overall, total WMH volume increased by approximately 97% over the follow‐up period, while total lesion volume showed an approximately fourfold increase. The largest relative changes were observed in periventricular regions, with approximately eightfold and twelvefold increases in WMH and lesion volumes. The global Fazekas score remained generally low at first 0.01 [0.42] and last 0.10 [0.58] brain MRI, and there was no statistically significant increase in follow‐up (*p* = 0.074).

Group comparisons with follow up images in WMH total volumes showed higher burden in women (*n* = 10) than in men (*n* = 5) (median 20.56 [50.14] vs. 7.14 [11.79]; *p* = 0.037) and higher global Fazekas score (median 0.56 [0.92] vs. 0.00 [0.096]; *p* = 0.031). Patients with the m.3243A>G variant (*n* = 7) had higher total WMH volume (22.87 [36.16] vs. 7.94 [6.83]; *p* = 0.015) and deep WMH volume (12.60 [27.34] vs. 2.24 [2.33]; *p* = 0.008) than those with other mitochondrial diagnoses (*n* = 8). No differences were found according to SLE history or brain symptoms. For lesions, women had higher total (17.60 [45.66] vs. 4.72 [7.82]; *p* = 0.050), juxtacortical (1.15 [1.90] vs. 0.16 [0.19]; *p* = 0.023) and deep WM (1.74 [1.71] vs. 0.25 [0.36]; *p* = 0.014) lesion volumes than men. Patients with m.3243A>G also had higher total lesions (15.40 [32.93] vs. 2.54 [15.37]; *p* = 0.049), periventricular (13.36 [26.91] vs. 1.67 [14.12]; *p* = 0.049) and deep WM lesion volumes (2.04 [2.11] vs. 0.29 [1.29]; *p* = 0.015). History of SLEs or brain symptoms was not associated with lesion volumes.

**FIGURE 2:**
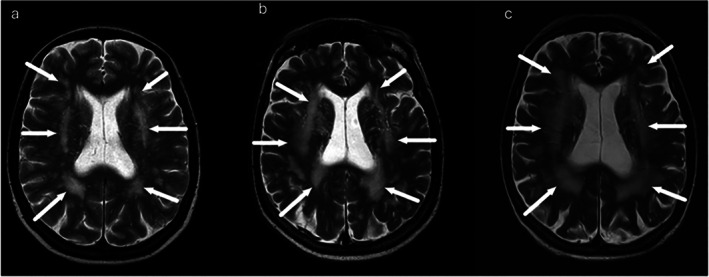
Progressive WMH in patient with the m.3243A>G variant. (a–c) Patient with the m.3243A>G variant: (a) T2 image, patient age 62 years; (b) T2 image, 72 years; (c) T2 image, 74 years.

Annualised progression analysis included 15 patients with available follow‐up imaging. WMH total volume increased by 1.23 [3.59] mL/year, by region deep WM 0.29 [2.08], periventricular 0.25 [0.52], juxtacortical 0.18 [0.99]. Infratentorial change was negligible. One‐sample Wilcoxon tests showed significant supratentorial progression (periventricular *p* = 0.009; juxtacortical *p* = 0.005; deep WM *p* = 0.023), with no change infratentorial. Lesion volume progressed by 1.47 [3.60] mL/year in total, periventricular 1.46 [2.73], juxtacortical 0.00085 [0.261], deep WM −0.011 [0.091]. Infratentorial change remained minimal. Figure 2 demonstrates the observed progression of WMH volumes in a patient with m.3243A>G variant. One‐sample Wilcoxon tests indicated significant progression in total and periventricular lesion volumes (both *p* < 0.001), with no evidence of change in other compartments (all *p* ≥ 0.273). Annual change in global Fazekas score was non‐significant (*p* = 0.096). Subgroup analyses showed no differences in WMH or lesion volumes, or in Fazekas score progression, by sex, genotype, or history of SLE or brain symptoms. In the m.3243A>G subgroup (*n* = 7, median follow‐up 5 years), WMH burden showed a non‐significant increase over time. Median annual WMH volume increase was 2.89 [3.97] ml/year in total WM, with contributions from deep (0.82 [3.20]), periventricular (0.25 [0.42]) and juxtacortical regions (0.48 [1.11]).

**TABLE 2:** Brain white matter hyperintensity volumes regionally in baseline and follow‐up MRI.

	Baseline median [IQR], *N* = 36	Follow‐up median [IQR], *N* = 15	Annualised change^a^	*p*
WMH total	6.37 [24.06]	12.57 [28.58]	1.23 [3.59]	< 0.001
Deep WMH	3.21 [16.41]	5.68 [26.85]	0.29 [2.08]	0.005
Periventricular WMH	0.50 [5.00]	4.41 [6.61]	0.25 [0.52]	0.003
Juxtacortical WMH	0.15 [0.63]	0.97 [2.81]	0.18 [0.99]	0.005
Fazekas	0.01 [0.42]	0.10 [0.58]	0.007 [0.08]	0.074

*Note:* The *p* values refer to Wilcoxon signed‐rank test comparing baseline and follow‐up measures.

Abbreviations: IQR = interquartile range, MRI = magnetic resonance imaging, WMH = white matter hyperintensity.

^a^ Annualised change was calculated as (follow‐up value − baseline value) divided by the individual follow‐up interval (years).

## 4 Discussion

We quantified WMH and lesion volumes and the global Fazekas score using the cNeuro MR tool in 36 predominantly adult patients with genetically confirmed mitochondrial disease, the m.3243A>G in mtDNA being the most common aetiology. Our results indicate that WM abnormalities are common and progressive in adult mitochondrial disease. In the general aging population, WMHs are most often attributable to CSVD, typically associated with chronic hypertension and other vascular risk factors such as diabetes, hyperlipidaemia and smoking [28, 29]. The median age at first MRI in our cohort was 49 years, which is younger than in most population‐based SVD cohorts where WMH typically emerge in late life [10, 30]. Traditional vascular risk factors were present in our cohort (Table 1) yet their contribution to WMH burden is likely limited, as these conditions may have been diagnosed later during follow‐up and were generally under good medical control given the long‐term clinical surveillance of this patient group. These findings are clinically relevant, as WM changes related to mitochondrial disease may be erroneously interpreted as common SVD, particularly in middle‐aged and older patients, potentially contributing to diagnostic uncertainty or delayed recognition of the underlying disorder.

At baseline, WMH burden was already evident in all supratentorial regions, most prominently in deep and periventricular WM, whereas infratentorial involvement was minimal. During follow‐up, WMH volume increased significantly in periventricular, deep and juxtacortical regions, and lesion progression was driven mainly by periventricular changes. In the general population, periventricular WMHs are reported to increase more rapidly than deep WMHs [31]. Among the patients with mitochondrial disease, deep WM showed the highest median annual progression in WMHs, but differences were small (0.29 vs. 0.25 mL/year), and there was substantial interindividual variability, limiting the ability to draw firm conclusions regarding region‐specific susceptibility.

WM injury in mitochondrial disease likely reflects overlapping vascular and metabolic mechanisms. While WM degeneration in the general population is associated with chronic hypoperfusion, oxidative stress and glial dysfunction [32, 33], mitochondrial disorders involve similar pathways that are activated earlier and more extensively due to intrinsic oxidative phosphorylation defects [11, 34]. This leads to ATP depletion, increased reactive oxygen species and impaired nitric oxide signalling, rendering oligodendrocytes, axons and endothelial cells particularly vulnerable [35].

In m.3243A>G disease, mitochondrial dysfunction may additionally contribute to microvascular injury and diffuse WM damage even in the absence of overt stroke‐like lesions [36, 37]. Notably, in our cohort, a history of SLE was not associated with increased WMH burden, despite its association with greater cortical atrophy. This suggests that SLLs primarily affect cortical and subcortical regions rather than driving persistent WM changes. Together, these findings support a model in which mitochondrial energy failure and secondary vascular stress contribute to progressive WM pathology that is distinct from, yet partially overlapping with, sporadic small‐vessel disease.

In the longitudinal subset, women showed higher WMH and lesion burden and higher Fazekas scores at follow‐up, although no differences were observed at baseline or in annual progression rates. Genotype also appeared to influence WM burden. Carriers of the m.3243A>G mutation showed greater total and deep WMH and lesion volumes compared to other mitochondrial diagnoses, consistent with the broader neurodegenerative and WM phenotype reported in m.3243A>G cohorts [6, 7, 35, 38]. This also suggests a disease‐specific contribution to WM injury. However, the modest cohort size limits robust genotype‐specific conclusions. Findings in patients with nuclear DNA–related disorders, including POLG and LBSL, were consistent with previously described typical imaging patterns [14, 15, 16, 18].

This study has several limitations related to its retrospective, single‐centre design. The modest sample size limits the statistical power of subgroup analyses and increases uncertainty in subgroup estimates. In particular, the small number of patients with nuclear gene variants necessitates cautious interpretation of findings in these subgroups. These results should therefore be considered primarily descriptive. However, these data were retained for completeness, given the clinical relevance of characteristic MRI patterns in conditions such as LBSL (DARS2) and POLG‐related disease.

As data on mtDNA variant heteroplasmy levels were not available for most patients, we were unable to assess whether heteroplasmy influences WMH burden or progression. Some analyses were also exploratory and not adjusted for multiple comparisons and should therefore be interpreted with caution. The use of multiple MR scanners (1.5 and 3 T) may have introduced measurement heterogeneity. Selection bias is possible, as only patients with MRI data suitable for analysis were included. Automated WMH segmentation methods are optimised for supratentorial pathology and may under‐detect focal infratentorial lesions, which are relevant for certain phenotypes such as POLG variants and LBSL. Although imaging measures were referenced to age‐ and sex‐adjusted normative data, residual confounding by age cannot be fully excluded in this retrospective cohort. The absence of an external control cohort limits the generalisability of the findings.

Key strengths of our study include a genetically confirmed mitochondrial cohort with systematic clinical phenotyping, quantitative MRI analyses from an FDA‐cleared, automated imaging tool (cNeuro) with intracranial‐volume normalisation, and region‐specific compartment analyses (periventricular, deep, juxtacortical) across 73 images. The data also included longitudinal follow‐up for 15 patients, with a median duration of 6 years. We combined semiquantitative (Fazekas) and volumetric metrics, enabling detection of subtle progression that visual scales may undercapture.

In this study, we demonstrate that patients with mitochondrial disease, particularly those with the m.3243A>G variant, develop an early, progressive burden of WM abnormalities that cannot be explained by conventional vascular risk factors alone. Neuropathological data support this interpretation by demonstrating that, in m.3243A>G disease, the most severe mitochondrial dysfunction affects leptomeningeal and cortical vessels rather than neurons [39]. This vascular involvement may help explain the diffuse WM injury seen in mitochondrial patients. Our data suggest that WM pathology accrues through disease‐specific mechanisms distinct from sporadic small‐vessel disease and may evolve in part independently of SLEs. By combining automated volumetric quantification with clinical and genetic characterisation, this study adds new evidence that mitochondrial disease contributes to a unique pattern of WM involvement at a relatively young age. These findings emphasise the importance of systematic MRI follow‐up and suggest that quantitative volumetric measures may provide sensitive indicators of disease progression beyond conventional visual scales. Advanced imaging approaches may further support earlier detection of progression, identification of high‐risk patients and monitoring of treatment response. Future studies with larger, prospective cohorts and standardised imaging protocols are warranted to validate these results, clarify sex‐ and genotype‐specific vulnerability and ultimately assess the prognostic value of WM progression for clinical outcomes.

## Author Contributions

All authors contributed to the study conception and design. Data collection was performed by N.M. and statistical analysis by N.M. and J.H. The first draft of the manuscript was written by N.M. All authors commented on previous versions of the manuscript. All authors read and approved the final manuscript.

## Funding

This work was supported by Turku University Hospital (Finnish Government Research Funding [VTR]; N.M.).

## Ethics Statement

This research was covered by the TUH research permission TO4/016/16.

## Consent

Individual informed consent was not required for this retrospective, register‐based study.

## Conflicts of Interest

The authors declare no conflicts of interest.

## Data Availability

The data that support the findings of this study are available from the corresponding author upon reasonable request.
